# Factors influencing readiness to deploy in disaster response: findings from a cross-sectional survey of the Department of Veterans Affairs Disaster Emergency Medical Personnel System

**DOI:** 10.1186/1471-227X-14-16

**Published:** 2014-07-19

**Authors:** Nicole K Zagelbaum, Kevin C Heslin, Judith A Stein, Josef Ruzek, Robert E Smith, Tam Nyugen, Aram Dobalian

**Affiliations:** 1 Veterans Emergency Management Evaluation Center, 16111 Plummer Street MS-152, North Hills, CA 91343, USA; 2 National Center for PTSD, 795 Willow Rd, Menlo Park, CA 94025, USA; 3 Veterans Health Administration Office of Emergency Management, 510 Butler Ave Bldg. 203B, Martinsburg, VA 25405, USA; 4 Department of Health Policy and Management, University of California, Los Angeles Fielding School of Public Health, 650 Charles Young Dr. S., Los Angeles, CA 90095, USA

**Keywords:** Emergency preparedness, Public health, Training, Readiness, First responder, Gender, Stress

## Abstract

**Background:**

The Disaster Emergency Medical Personnel System (DEMPS) program provides a system of volunteers whereby active or retired Department of Veterans Affairs (VA) personnel can register to be deployed to support other VA facilities or the nation during national emergencies or disasters. Both early and ongoing volunteer training is required to participate.

**Methods:**

This study aims to identify factors that impact willingness to deploy in the event of an emergency. This analysis was based on responses from 2,385 survey respondents (response rate, 29%). Latent variable path models were developed and tested using the EQS structural equations modeling program. Background demographic variables of education, age, minority ethnicity, and female gender were used as predictors of intervening latent variables of *DEMPS Volunteer Experience*, *Positive Attitude about Training*, and *Stress*. The model had acceptable fit statistics, and all three intermediate latent variables significantly predicted the outcome latent variable *Readiness to Deploy*.

**Results:**

*DEMPS Volunteer Experience* and a *Positive Attitude about Training* were associated with *Readiness to Deploy. Stress* was associated with decreased *Readiness to Deploy*. Female gender was negatively correlated with *Readiness to Deploy*; however, there was an indirect relationship between female gender and *Readiness to Deploy* through *Positive Attitude about Training*.

**Conclusions:**

These findings suggest that volunteer emergency management response programs such as DEMPS should consider how best to address the factors that may make women less ready to deploy than men in order to ensure adequate gender representation among emergency responders. The findings underscore the importance of training opportunities to ensure that gender-sensitive support is a strong component of emergency response, and may apply to other emergency response programs such as the Medical Reserve Corps and the American Red Cross.

## Background

Organizational training programs for emergency responders are critical to quickly identify and deploy appropriate resources in the overall response effort [1]. Results from several studies suggest that training is linked with greater effectiveness of emergency response efforts and that training of health personnel is positively associated with willingness to volunteer and deploy in the event of a disaster [2-4]. However, there exist several significant gaps in training for emergency providers. For example, an Institute of Medicine report found that only 49% of hospitals trained their residents and interns in disaster preparedness, which represents a national point of concern in the emergency management community [5]. A number of studies suggest that willingness to report to work could be a substantial problem within the disaster response workforce, depending on the type of event. Several large surveys have found that the percent of healthcare workers willing to report to work during disasters and emergencies can be 50% or less for severe snow storms [6] and influenza pandemics [7,8], and less than 60% for chemical or radiation events [9], leading to potentially crippling personnel shortage in times of great need. It is important to recognize the factors that impact an appropriate medical response to a disaster in a global context. A survey of over 900 nurses in China found that as few as one-third were willing to report to work during an infectious disease outbreak and also found that the most significant factor affecting to willingness to work was clinical experience [10]. Another survey of primary care health staff in Guangdong Province, China found that only one-fourth of respondents had participated in emergency response in the past, reflecting both poor response capacity and lack of experience [11]. In a survey of Israeli nurses, less than half had reported to work when asked in the past year following a response [12]. Another Israeli survey indicated that only 51% of hospital workers said they would report following a missile attack [13]. Finally, in a survey of hospital workers in the United Kingdom, the average likelihood of reporting to work in the event of an influenza pandemic was 59.3%; rates varied by factors such as familial support and previous disaster training [14].

Underscoring the critical role of knowledge and skills in competent disaster response work, the World Association for Disaster and Emergency Medicine [15] and other organizations have recommended guidelines for education and training programs for disaster medicine and humanitarian aid. The need for these recommendations is supported by work from around the globe. For example, among Australian emergency prehospital health care providers, less than 5% of employees felt they had been adequately trained concerning avian influenza [16]. Another article from Australia reported that willingness to work during a pandemic was associated with increased knowledge, education, and training about infectious agents among healthcare workers [17].

Limited work exists on the role of gender in disaster response readiness – i.e. the ability and willingness of responders of both genders to deploy and perform assigned duties [18]. One survey of healthcare workers found that women were less willing to report to duty in the event of a disaster [6]. An evaluation of Veterans found that women report stress to a greater extent than do men following similar levels of exposure to deployment stressors [19]. Gender differences in psychological stress after disasters are also important to note among emergency responders, as this could impact the work performance of deployed personnel [20].

Disasters and public health emergencies increase the general demand not only for emergency services and basic health care, but also for a wide range of gender-specific health and social services [21]. For example, in the weeks after an 8.0-magnitude earthquake in China, women reported markedly increased symptoms of lower genital tract infection, pelvic inflammatory disease, and menstrual disorders [22]. After the 2001 attacks on the World Trade Center, women were more likely to report using psychiatric medications than were men [23]. Women responders may be able to communicate and interact with women with disaster-related distress or gender-specific health and social needs more effectively than male responders. For example, after responses by the police to domestic violence calls, female officers receive more positive evaluation than do male officers from battered women by providing increased empathy, referral services, and legal information [24,25]. In a survey of over 800 patients visiting the emergency department, women reported significantly more satisfaction with female physicians due to the increased amount of concern, trust, and overall rating of the experience [26]. These findings on specific needs of women during disasters and public health emergencies underscore the importance of ensuring adequate inclusion of women in the disaster response workforce. Given the need for gender-sensitive services in disaster response, as well as the evidence on the importance of gender-concordant service providers for women, the lack of work on the impact of gender on response readiness represents a significant gap in the literature on disaster preparedness.

Using survey data collected from a volunteer emergency management workforce organized by the Department of Veterans Affairs (VA), we tested the hypothesis that background demographic characteristics of education, age, ethnicity, and gender impacted intermediate variables of experience, attitudes about deployment, and stress. These variables were, in turn, hypothesized to predict readiness to deploy. The intermediate variables and outcome variable were constructed as multiple indicator latent variables. We were particularly interested in examining the relationship between gender and readiness to deploy in response to a disaster.

## Methods

The current literature on attitudes toward training lacks established, well-developed conceptual models [27]. Specifically, the relationship between background characteristics, organizational training, and readiness to deploy has not been well explored. Accordingly, we propose a conceptual framework that incorporates these elements. The model builds on Bloom’s “taxonomy of learning domains,” which incorporates three domains of educational activities as Knowledge, Skills, and Attitude (KSA) and has been used as a tool to measure educational interventions in nursing personnel, among many others [28-30]. Francke and colleagues’ conceptual model incorporates KSAs to measure the determinants for behavioral change subsequent to continuing education programs based on the interplay between background characteristics of the individual and the educational program [27]. It has been identified in nursing competencies that ongoing development of knowledge and clinical expertise helps to build skills within the KSA model [31]. In the current study, a number of questionnaire items are key indicators of the KSA domains of Bloom’s model. For example, agreement with the item, “DEMPS training provided useful information about my role and responsibilities during deployment” likely reflects a level of knowledge about the requirements of deployment. Agreement with the item “I am confident in my ability to provide quality care during deployment” is an indicator for an attitude of confidence or self-efficacy that is important to successful performance in disaster response.

In our adapted conceptual model on disaster education and attitudes toward training, background characteristics and experience as a DEMPS volunteer directly influence KSAs. This, in turn, impacts readiness to deploy. Age and ethnicity were also included as background demographic variables. In addition, the proposed model includes stress as an intermediate variable, as Ejaz’s conceptual model specifies background characteristics and stress as determinants of job satisfaction in direct care workers [32].

The respondent sample for this study consists of volunteers in the DEMPS program, a system of volunteers and training by which active or retired VA personnel can register for deployment for internal or external support as may be requested by federal agencies [33]. DEMPS volunteers include mental health personnel, healthcare support professionals, physicians, nurses, physician assistants, pharmacy technicians, allied health professionals, and allied support such as cooks and drivers [33].

The entire DEMPS workforce of 8,250 volunteers was invited to participate in the online survey. On October 16, 2011, an initial email invitation was sent to the DEMPS workforce and three subsequent reminder emails were sent to those who did not respond to the initial survey invitation. Each follow-up email was sent one week apart. Of those invited, 2,385 volunteers responded to the survey for a total response rate of 29%. Because of item-level nonresponse, 2079 individuals had usable data for this analysis. The study protocol was reviewed and approved by the Stanford University Institutional Review Board (IRB).

### Measures

#### Background sociodemographic characteristics

*Education* was based on a 1–5 scale (1 = high school diploma or equivalent, 2 = Bachelor’s degree, 3 = Master’s degree, 4 = Doctoral degree, 5 = Medical degree). The median degree was a Master’s degree and the modal response was a medical degree. *Age* was measured on a 1–6 scale (1 = 18–25, 2 = 26–30, 3 = 31–40, 4 = 41–50, 5 = 51–60, 6 = >60). The modal age range was ages 41–50. The median age range was ages 31–40. *Ethnicity* in the study was represented by minority ethnicity (21%) vs. White ethnicity (79%), coded 1 or 0 respectively. Minority ethnicity included American Indian/Alaskan native (2%), Asian or Pacific Islander (2%), African-American (11%), and Hispanic (6%). The sample was 55% female; gender was scored male = 1, female = 2. The top three occupations were “administrative, technical, or professional employee” (n = 246); Registered nurse, Level II (n = 236), and “Other administrative, technical, professional, or clerical worker” (n = 233) (not shown in table).

#### Intermediate latent variables

*Experienced DEMPS Volunteer* was indicated by two items: 1) How long have you been a DEMPS volunteer? (1 = less than 1 year; 2 = 1–2 years; 3 = 3–5 years, 4 = >5 years). 2) Have you ever been deployed as a DEMPS volunteer to a disaster site? (1 = no; 2 = yes).

*Positive Attitude about Training* was indicated by 9 items scaled 1–7 that ranged from “strongly disagree” to “strongly agree.” Typical items included “The training I received was appropriate given DEMPS mission and goals” and “My DEMPS-related training events and exercises were realistic.” Table 1 includes the wording for all of the items in this scale.

**Table 1:** Questionnaire on DEMPS training experiences

**These next questions are about your DEMPS training experiences as a whole. Please select one response to indicate how much you disagree or agree with each of the following statements:**							
	**Strongly disagree**	**Disagree**	**Slightly disagree**	**Neither agree nor disagree**	**Slightly agree**	**Agree**	**Strongly agree**
The training I received was appropriate given DEMPS mission and goals.	1	2	3	4	5	6	7
My DEMPS-related training events and exercises were realistic.	1	2	3	4	5	6	7
DEMPS training events and exercises were well organized.	1	2	3	4	5	6	7
DEMPS training events and exercises were good learning opportunities.	1	2	3	4	5	6	7
DEMPS training prepared me about what to expect during deployment.	1	2	3	4	5	6	7
DEMPS training provided useful information about my role and responsibilities during deployment.	1	2	3	4	5	6	7
I have been provided with sufficient training in preparation for deployment.	1	2	3	4	5	6	7
I plan to continue to volunteer with DEMPS for at least another year.	1	2	3	4	5	6	7
I would recommend volunteering with DEMPS to others.	1	2	3	4	5	6	7
All in all, I am satisfied with my training experience(s) with DEMPS.	1	2	3	4	5	6	7

*Stress* was assessed with 8 items from the Perceived Stress Scale [34]. Items are scaled from 0 (never) to 4 (very often). Typical items included: “How often have you been upset because of something that happened unexpectedly?”, and “How often have you felt that you were unable to control the important things in your life?”

#### Outcome latent variable

*Readiness to Deploy* was indicated by 9 items scaled from 1 (strongly disagree) to 7 (strongly agree). Typical items included: “I am prepared for deployment” and “I am confident in my ability to provide quality care during deployment.” Table 2 includes the wording for all of the items in this scale.

**Table 2:** Questionnaire on perceived readiness and preparation

**These next questions are about your level of readiness and preparation for deployment. Please select one response to indicate how much you disagree or agree with each of the following statements:**							
	**Strongly disagree**	**Disagree**	**Slightly disagree**	**Neither agree nor disagree**	**Slightly agree**	**Agree**	**Strongly agree**
I am prepared for deployment.	1	2	3	4	5	6	7
My family and/or friends support my participation as a DEMPS volunteer.	1	2	3	4	5	6	7
My supervisors and coworkers support my participation as a DEMPS volunteer.	1	2	3	4	5	6	7
I am confident in my ability to effectively respond to the deployment mobilization process.	1	2	3	4	5	6	7
I am confident in my ability to meet all administrative demands during demobilization.	1	2	3	4	5	6	7
I am confident in my ability to provide quality care during deployment.	1	2	3	4	5	6	7
I am confident in my ability to manage stress during deployment.	1	2	3	4	5	6	7
I am confident that I will know how to access mental health support if needed while deployed.	1	2	3	4	5	6	7
I feel prepared to deal with unexpected situations that may occur during deployment.	1	2	3	4	5	6	7
If an event was to occur in the next 3 months, and I am asked to deploy, I am likely to go.	1	2	3	4	5	6	7

### Analysis

Measurement and path models were developed and tested with a latent variable covariance structure analysis using the EQS structural equations modeling program [35]. Goodness-of-fit was assessed with the Maximum-Likelihood χ^2^ statistic (ML χ^2^), the Comparative Fit Index (CFI), the Satorra-Bentler χ^2^ (S-B χ^2^), the Robust Comparative Fit Index (RCFI), and the Root Mean Squared Error of Approximation (RMSEA) [36,37]. The S-B χ^2^ was used in addition to the ML χ^2^ because it is more appropriate and robust when the data depart from multivariate normality as the data did in this study (Mardia’s normalized estimate = 139.76). The CFI and RCFI range from 0 to 1 and reflect the improvement in fit of a hypothesized model over a model of complete independence among the measured variables. Values at .95 or greater are desirable, indicating that the hypothesized model reproduces 95% or more of the covariation in the data [37]. The RMSEA is a measure of fit per degrees of freedom, controlling for sample size, and values less than .06 indicate a relatively good fit between the hypothesized model and the observed data [37].

An initial Confirmatory Factor Analysis (CFA) assessed the adequacy of the hypothesized measurement model and provided bivariate correlations among the model variables. Several items within the scales were similar, and we expected that some additional associations would be needed for fit improvement. Suggestions from the LaGrange Multiplier (LM) test for additional relationships between the error residuals within the scales were evaluated and were allowed if they made sense theoretically and logically [38]. The LM test reports supplementary modifications to the original model that will improve fit. We then tested a predictive path model that positioned the background demographic variables of education, age, minority ethnicity, and female gender as predictors of intervening variables of *Experienced DEMPS Volunteer*, *Positive Attitude about Training*, and *Stress*. In turn, all background variables predicted *Readiness to Deploy*. Correlations were allowed among the predictive background variables if they were significant. Non-significant paths and correlations in this model were trimmed gradually following the recommended procedure of MacCallum [39]. In addition to direct paths, we also examined indirect paths mediated through the intermediate latent variables.

### Missing data

About 10% of the sample was missing 1 or more individual items. To ascertain that the data were Missing Completely At Random (MCAR), the Full Information Maximum Likelihood (FIML) missing data method available in EQS that uses an EM algorithm was employed [36]. In EM imputation, parameter estimates are obtained by iterating an expectation (E) step and a maximization (M) step. FIML is the recommended data imputation method when using the EQS structural equations modeling program. Diagnostics using the models described below indicated that the missing data points were MCAR. Further, results were the same with both methods.

## Results

### Confirmatory factor analysis

The final CFA had an excellent fit: maximum-likelihood solution: ML χ^2^ = 1874.29, 443 *df;* CFI = .96, RMSEA = .046, Robust S-B χ^2^ = 1545.91, 443 *df;* RCFI = .95; RMSEA = .040. All hypothesized factor loadings were significant (*p* ≤ .001). Table 3 presents the factor loadings, means, and standard deviations of the measured variables. It is notable that scores on the *Positive Attitude about Training* and *Readiness to Deploy* measures were relatively high, whereas scores on *Stress* were low. Table 4 reports the correlations among all of the latent variables and the demographic single-item variables in the CFA. A few correlated error residuals were added to the CFA model to improve the fit. Examining the larger correlations (all *p* ≤ .001) we note that older participants reported more experience (.24), a more positive attitude about training (.10), and more Readiness to Deploy (.09). Female gender was only minimally but in some cases significantly associated with the other variables in the model (greater education, .09). Those that were experienced reported more positive attitudes (.14) and greater Readiness to Deploy (.25). Stress was associated with a less positive attitude (−.09) and less Readiness to Deploy (−.21). A positive attitude about training was highly associated with Readiness to Deploy (.47).

**Table 3:** **Summary statistics and factor loadings of measured variables in the confirmatory factor analysis (*N *** **= 2079)**

**Measured demographic variables**	**Mean (S.D). or %**	**Factor loading**^**a**^
Education (range = 1–5)	3.01 (1.44)	NA
Age (1–6)	3.30 (0.98)	NA
Minority	21%	NA
Female	55%	NA
**Latent variables**		
Experienced DEMPS volunteer		
Length of time volunteer (1–4)	2.65 (0.97)	.75
Ever deployed to disaster site	18%	.64
Positive attitude about training (1–7)		
Training appropriate	5.07 (1.55)	.90
Training realistic	4.74 (1.55)	.86
Well organized	4.79 (1.50)	.87
Good learning opportunities	4.92 (1.53)	.88
Prepared what to expect	4.90 (1.59)	.90
Useful information about roles	4.94 (1.61)	.89
Training sufficient	4.55 (1.81)	.85
Recommend volunteering	5.75 (1.42)	.59
Satisfied with training	4.84 (1.81)	.89
Stress (0–4)		
Upset at unexpected	1.31 (0.70)	.66
Unable to control important things	1.18 (0.79)	.66
Felt nervous and “stressed”	1.49 (0.74)	.69
Could not cope	0.88 (0.77)	.63
Hard to control irritations	1.36 (1.05)	.42
Felt you were on top of things (R)	0.66 (0.77)	.37
Angered by lack of control	1.25 (0.75)	.70
Difficulties piling up	0.89 (0.71)	.73
Readiness to deploy (1–7)		
I am prepared for deployment	5.46 (1.67)	.79
I have family/friend support	6.08 (1.17)	.59
Support from supervisors	5.09 (1.79)	.37
Confident in my ability to respond	5.90 (1.38)	.90
Confident - administrative demands	5.89 (1.39)	.88
Confident – provide quality care	6.20 (1.16)	.81
Confident - access mental health	5.84 (1.47)	.80
Confident – deal with unexpected	5.99 (1.33)	.86
Likely to go if needed next 3 months	6.06 (1.37)	.62

^a^All factor loadings significant, p ≤ .001. NA = not applicable. R = reverse scored.

**Table 4:** **Correlations among variables in model (*N =*** **2079)**

	**1**	**2**	**3**	**4**	**5**	**6**	**7**	**8**
1. Education	—							
2. Age	.08**	—						
3. Female gender	.09**	-.05	—					
4. Minority	-.06*	.03	-.06*	—				
5. Experienced volunteer	.00	.24**	-.01	-.05	—			
6. Positive attitude	.01	.10**	.06*	.01	.14**	—		
7. Stress	-.04	-.03	.01	.04	-.01	-.09**	—	
8. Readiness to deploy	-.02	.09**	-.06*	.02	.25**	.47**	-.21**	—

*p ≤ .05, ** p ≤ .001.

### Path analysis

**Figure 1:**
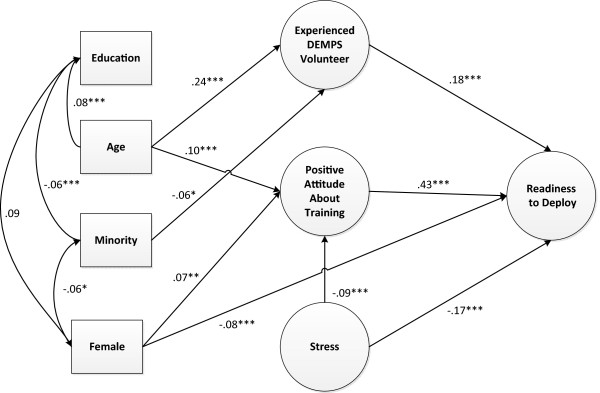
**Model depicting significant regression paths that predict *Readiness to Deploy *among 2079 DEMPS volunteers.** Large circles represent latent variables; rectangles represent single-item indicators. 1-headed arrows represent regression coefficients; 2-headed arrows represent correlations. Regression coefficients are standardized. (*= p ≤ .05, **= p ≤ .01, ***= p ≤ .001).

Figure 1 presents the final path model after trimming. This model has acceptable fit statistics: ML χ^2^ = 1886.32, 447 *df;* CFI = .96, RMSEA = .045, Robust S-B χ^2^ = 1563.41, 447 *df;* RCFI = .95; RMSEA = .039. All three intermediate latent variables (*Experienced DEMPS Volunteer*, *Positive Attitude about Training*, and *Stress*) significantly predicted the outcome latent variable *Readiness to Deploy*. In addition, female gender independently predicted less Readiness to Deploy (*p* ≤ .001). There were also significant indirect effects on Readiness to Deploy by age (*p* ≤ .001), female gender (*p* ≤ .01), and stress (*p* ≤ .001). The indirect effect of female gender was positive (mediated through *Positive Attitude about Training*) although the direct effect was negative.

To assess the representativeness of the respondents to the DEMPS survey, we obtained data on demographic characteristics of the VA workforce from the 2013 VA All-Employee Survey [40]. Twenty-one percent of the respondents to the DEMPS survey were of racial/ethnic minority backgrounds, compared with 33% of the overall VA workforce. Fifty-five percent of DEMPS survey respondents were female, compared with 59% of the VA workforce. The modal age category for the DEMPS survey respondents was 41–50 years old, compared with 50–59 years old in the VA workforce.

## Discussion

Recruiting and retaining volunteers who are ready to deploy is essential to mounting an effective response during an emergency or disaster. Training in disaster medicine has a direct impact on a healthcare professional’s ability to be useful during an emergency [1]. Training is highly desired among emergency responders, a vast majority of whom report they would benefit from additional training activities [41]. As hypothesized and in accord with our conceptual model, the current study found that age was positively associated with *Experience as a DEMPS volunteer*, *Positive Attitude about Training*, and *Readiness to Deploy*. This finding is also consistent with the notion that more trained and experienced healthcare workers are more likely to feel prepared to deploy [7]. Accordingly, our findings suggest that inexperienced responders may benefit from additional training in order to increase their readiness to deploy. There was also positive association between *Positive Attitude about Training* and *Readiness to Deploy*. Measuring attitudes about training is important for emergency organizations to design better training to ensure a robust emergency response workforce.

The current study found that female gender is negatively correlated with *Readiness to Deploy*. This finding is consistent with previous studies, where related factors such as familial obligations, marital status, and spousal support during deployment have been postulated to play a significant role in overall willingness to deploy for military service [42]. Given the literature suggesting that gender is an important component of cultural competence that would allow emergency responders to effectively engage with the diverse population that may be impacted by a disaster, it is important that women be included in emergency response groups [21,26]. As such, our finding that female gender has a modest negative correlation with *Readiness to Deploy* suggests that systems such as DEMPS should consider how best to address the factors that may make women less ready to deploy than men in order to ensure adequate gender representation among emergency responders. To the extent that at least some of the gender differential relates to familial obligations, marital status, and spousal support, policies that address these concerns would also benefit male responders.

The model also identified an indirect positive relationship between female gender and *Readiness to Deploy* mediated by *Positive Attitudes toward Training*. This finding is consistent with the presented conceptual model where gender influences the KSAs that will ultimately impact healthcare workers’ readiness to deploy. Our study also found a positive correlation between female gender and having a *Positive Attitude about Training*, whereas those with less positive attitudes were less ready to deploy. This finding highlights the importance of providing ongoing training support to disaster response personnel to ensure the ability to provide gender-sensitive care during an emergency. A better understanding of gender differences in readiness could inform the design and implementation of emergency management training activities. This represents another key point of interest for VA and other emergency response organizations, where efforts to increase access to training and support for female responders could lead to an overall more prepared female deployment staff.

The analysis showed that individuals who reported stress were less likely to report a positive attitude about training. This has implications for the DEMPS program, as stress has been linked to impaired work performance among military personnel, which may impact outcomes in daily occupational duties as well as activities during deployment [20]. When examining gender differences in stress, we expected women to report greater stress than men, because, in general, women are more likely to report stress, experience the physical and emotional symptoms of stress, as well as undergo increased stress over the past five years [43]. However, we did not find a correlation between gender and stress. This may be due to the fact that the DEMPS volunteer workforce generally represents a self-selected group of resilient women who are highly experienced in emergency management and disaster response work.

This study has limitations. About 10% of the sample had missing data. However, statistical analysis suggested that the missing data were missing completely at random (MCAR). Therefore, the events that led to any particular item being missing appear to be independent both of the observed variables and of unobserved parameters. In addition, the data were self-reported. Thus, *Readiness to Deploy* is based on an individual’s perceptions rather than objective standards. Nonetheless, it is likely that an individual’s readiness is largely influenced by the degree to which he or she believes they are able to deploy at a given point in time. The categories for the ordinal age variable were not of equal width. Age was not collected as a continuous variable, so we were unable to re-categorize it into intervals of equal length. Still, ordinal variables do not need to have equally sized categories in order to be useful analytically. We included the variable on “length of time” with DEMPS based on the notion that respondents registered with DEMPS for longer time periods would likely be exposed to both more training opportunities and more deployment opportunities; however, we realize that “length of time” is only a proxy measure of these variables. The 29% response rate is somewhat low; however, since the entire DEMPS workforce was included in the survey it is possible that nonresponders have no experience being deployed. It should be noted that we do not know whether measures of willingness to deploy in this or other studies actually predict future deployment behaviors. In addition, our study did not include measures of familial obligations, marital status, or spousal support. It is possible that the observed difference in *Readiness to Deploy* between the genders could be explained by such measures, had we been able to include these items in the study. Future work should assess the predictive validity of willingness to deploy measures, as well as the mechanisms by which gender impacts *Readiness to Deploy* by including measures of these factors.

## Conclusion

As previously discussed, there is a limited body of research that has explored the relationship between personnel support programs and readiness. During large scale disasters and emergencies, there is a need to identify and mobilize personnel who require the training and skills for appropriate response [33,44]. Understanding the significance of providing training to DEMPS volunteers has several simultaneous benefits. First, it provides ongoing support for volunteers to be able to provide excellent care in the event of a disaster. Second, it addresses a historical disparity in female healthcare providers who can respond to the gender-sensitive demands for a historically underserved demographic. The DEMPS volunteer program addresses an important need to identify and deploy trained health personnel in order to provide an effective response and comply with the VA’s emergency management mission [45]. Our findings regarding readiness to deploy may also apply to other emergency response programs such as the Medical Reserve Corps and the American Red Cross. Future work should explore similar relationships among these groups.

### Consent

Informed consent was obtained from the respondents for the publication of this report.

## Abbreviations

DEMPS: Disaster Emergency Medical Personnel System; VA: Department of Veterans Affairs; KSA: Knowledge, skills, and abilities; IRB: Internal review board; CFI: Comparative fit index; RCFI: Robust comparative fit index; RMSEA: Root mean squared error of approximation; CFA: Confirmatory factor analysis; LM: LaGrange multiplier; MCAR: Missing completely at random; FIML: Full information maximum likelihood.

## Competing interests

The authors declare that they have no competing interests.

## Authors’ contributions

KH and NZ participated in the conception and design of the study and wrote the manuscript. KH/AD/JR/TN participated in the design of the study and data acquisition. JS was responsible for data analysis and wrote the methods section. RS and TN participated in the design of the study and revision of the manuscript. All authors have read and approved the final version of the manuscript.

## Pre-publication history

The pre-publication history for this paper can be accessed here:

http://www.biomedcentral.com/1471-227X/14/16/prepub
